# Supplementary material for: Drifting discrete Jovian radio bursts reveal acceleration processes related to Ganymede and the main aurora
Source: Nat Commun. 2023 Oct 3;14:5981. doi: 10.1038/s41467-023-41617-8 (PMC10547699; doi:10.1038/s41467-023-41617-8)
Supplement: Supplementary file 1 — Supplementary Information [file 41467_2023_41617_MOESM1_ESM.pdf]

Supplementary information for :
Drifting discrete Jovian radio bursts reveal acceleration processes related to Ganymede and the main aurora

1 Positive slopes

For a significant fraction of the processed dynamic spectra (5 to 10%, see Table 1 in the article), positive slopes are found. We have checked visually all the corresponding cases with $SNR \geq 5$, and we have found that none of them actually corresponds to real positively drifting bursts. All consist of dynamic spectra devoid of Jupiter signal and entirely dominated by RFI. These dynamic spectra are not completely cleaned by the RFI mitigation steps, and some of them accidentally result in detections with positive slopes very close to the vertical or horizontal axis $\pm 15°$. Figure 1 displays a representative example of such a positive slope detected by the pipeline.

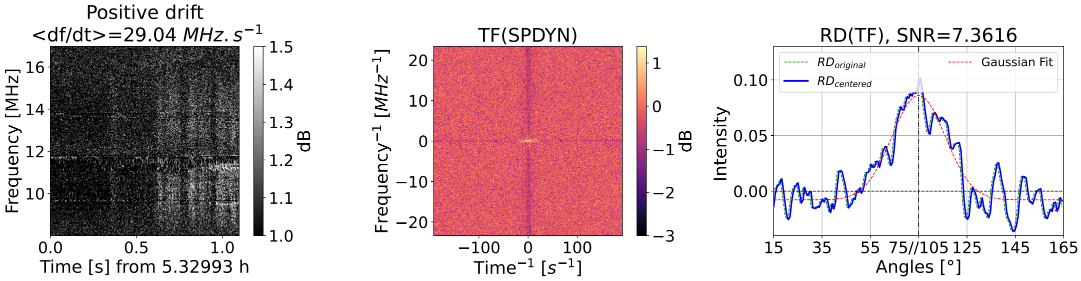

Supplementary Figure 1: **Example of a spurious detection of signals with a positive slope.** The dynamic spectrum (left panel) is dominated with weak vertical RFI that could not be completely removed by the RFI mitigation step of the processing. This results in a very intense horizontal line in the 2D-FFT (middle panel), which is not completely eliminated by the contrast enhancement procedure. As a result, a peak is detected with an SNR ≥ 7 in the Radon transform (rightside panel) very near 105°, i.e. close to the horizontal axis but on the side of positive slopes.

2 SNR distribution per type of emission and slope interval

Figure 2 shows, for each type of emission (Io-induced, Ganymede-induced, Main aurora) detected in LH and RH circular polarization, the distribution of SNR for the three intervals of drift-rates listed in Table 1 and displayed in Figure 3 in the article. Positive drifts are clearly associated with lower SNR in all cases, consistent with weak residual RFI. They are much less numerous than negative (real) drifts in Fig 2a,b (Io-induced case), where the largest SNR values are associated with faster drift-rates <-10 MHz/s. Positive drifts are less numerous in Fig 2c (Ganymede-induced, LH), but dominant in Fig 2d (Ganymede-induced, RH). This can be explained by the fact that for the time intervals that we analyzed, most of the Ganymede-induced emissions were LH polarized. For Ganymede, the faster negative drifts ($df/dt < -10$ MHz/s) are less numerous than the slower ones ($-10 \leq df/dt < 0$ MHz/s), but they are detected with slightly larger SNR. Drifting bursts associated with the main aurora (Fig 2e,f) display the same general behaviour as Ganymede-induced emissions, except that in this case most of the detected emissions were RH polarized.

3 Context observations

A further confirmation of the origin of our detected bursts is provided by the overall morphology of the emission around the time of detection of S-bursts of each type (Io-induced, Ganymede-induced

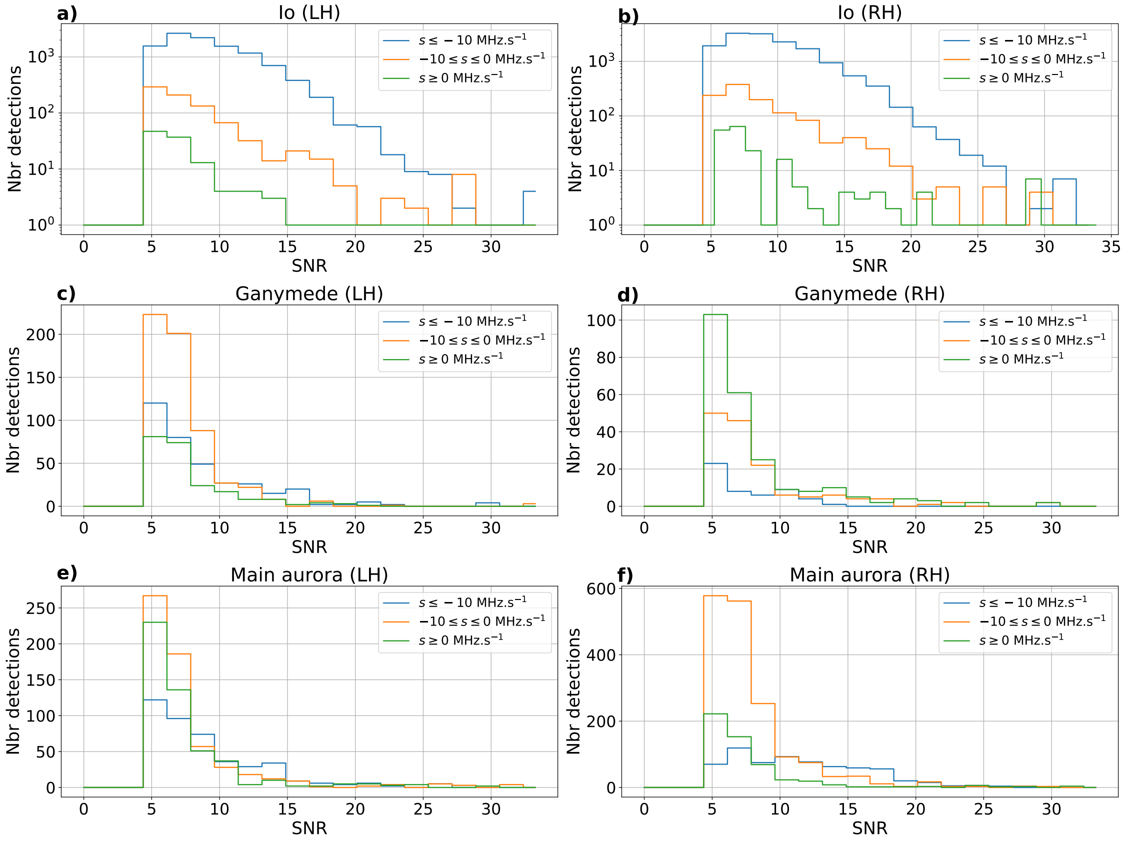

Supplementary Figure 2: **Histograms of SNR values for the three types of emissions identified and two polarizations.** The same intervals of drift-rate values as in Table 1 in the article are used, and the same color codes as in Figure 3 in the article. See text for details.

and Main aurora). The data were recorded by the NewRoutine digital receiver also connected to the NDA. This receiver observes Jupiter for longer periods of time than JunoN (8 hours per day) and provides thus complementary observations with high SNR but lower resolutions than JunoN [2, 1]. These context dynamic spectra, plotted with a compressed timescale (1-3 hours on Figure 3a-c), reveal the overall arc-shaped structures in which we detected S-bursts with JunoN. Their sense of curvature, polarization, spectral range, and time of occurrence can be compared to simulation results for identifying their origin [3]. The geometry at the time of each observation is indicated by the color lines on the CML–ϕ_{Io} and CML–$\phi_{Ganymede}$ maps of Figure 3d,e (CML = observer's longitude, ϕ_{Io} = orbital phase of Io, $\phi_{Ganymede}$ = orbital phase of Ganymede). Both approaches consistently indicate that panels a,b and c of Figure 3 correspond respectively to Io-C, Ganymede-C, and non-Io/non-Ganymede (i.e. main aurora) Jovian decameter emissions.

4 Presence of O mode emission ?

Figure 4 displays the distribution of drift rates per circular polarization only for the bursts detected in the Ganymede "C" box of Figure 1b in main text. These are the only bursts unambiguously related to Ganymede (bursts in box "B" are actually related to Io, and box "D" is defined with less well confidence [4]). Ganymede "C" bursts come from Jupiter's southern hemisphere, and should thus have LH circular polarization if emitted on the X mode. The left panel of Figure 4 indeed contains the largest number of bursts, of LH polarization, with both fast and slow negative drifts, thus slow drifting bursts cannot be explained by O mode only. But the right panel shows a dominant peak for slow drifts, in RH polarization, consistent with O mode emission produced by <1 keV electrons. It suggests that O mode bursts may indeed be present. Further detailed statistics on the polarization and slopes of a larger number of bursts sorted by emission type (Io- or Ganymede- A, B, C, D) should allow to study in details the emission mode and its relation with the bursts drifts. This provides a powerful means to characterize the electron cyclotron maser efficiency for various electron energies.

References

[1] L. Lamy, G. Kenfack, A. Duchêne, B. Cecconi, A. Loh, P. Zarka, C. Viou, P. Renaud, L. Denis, and A. Coffre. Nançay Decameter Array (NDA) Jupiter NewRoutine data collection, 2022.

[2] L. Lamy, P. Zarka, B. Cecconi, L. Klein, S. Masson, L. Denis, A. Coffre, and C. Viou. 1977-2017: 40 years of decametric observations of Jupiter and the Sun with the Nancay Decameter Array. In G. Fischer, G. Mann, M. Panchenko, and P. Zarka, editors, *Planetary Radio Emissions VIII*, pages 455–466, Jan. 2017.

[3] C. K. Louis, S. L. G. Hess, B. Cecconi, P. Zarka, L. Lamy, S. Aicardi, and A. Loh. ExPRES: an Exoplanetary and Planetary Radio Emissions Simulator. *Astronomy & Astrophysics*, 627:A30, July 2019.

[4] P. Zarka, M. S. Marques, C. Louis, V. B. Ryabov, L. Lamy, E. Echer, and B. Cecconi. Jupiter radio emission induced by Ganymede and consequences for the radio detection of exoplanets. *Astronomy & Astrophysics*, 618:A84, Oct. 2018.

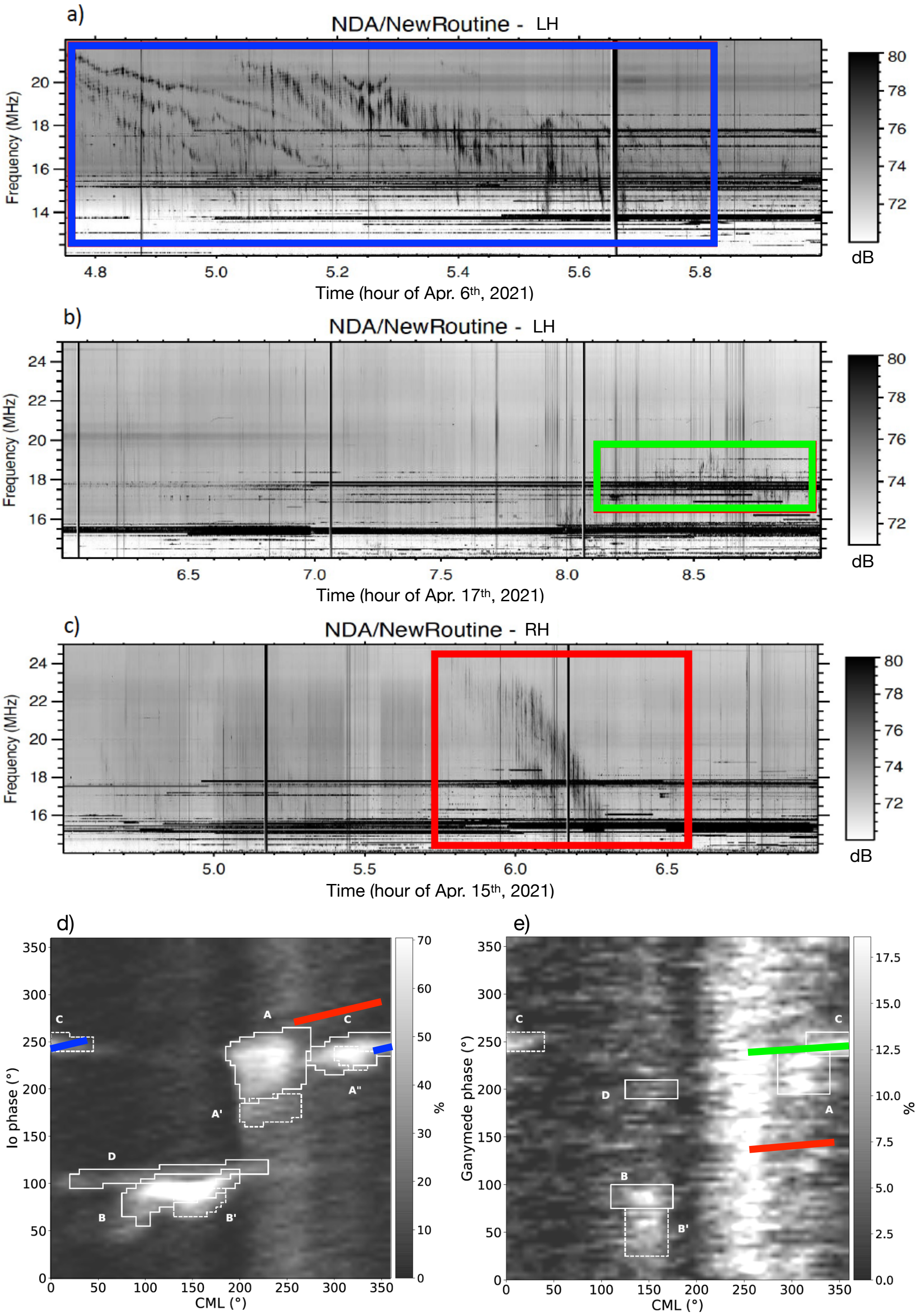

Supplementary Figure 3: (**a,b,c**) **Dynamic spectra recorded with the NDA/NewRoutine digital receiver** These dynamic spectra have a time-frequency resolution of ~0.5 s × 49 kHz. They reveal arc-shaped emissions lasting for a few hours (within the color boxes), the origin of which can be identified by comparison with the probability maps of panels (**d,e**) and/or simulation results [3]. Panel **a** displays an observation on April 6th, 2021, with a typical vertex-late (closed parenthesis) LH polarized Io-C arc. Panel **b**, from April 17th, 2021, displays a similar but weaker vertex-late LH polarized arc consistent with a Ganymede-C emission. Panel **c**, recorded on April 15th, 2021, displays a vertex-late RH polarized double arc that can be attributed neither to Io nor to Ganymede, and is therefore most likely related to the main aurora. Probability maps CML–ϕ_{Io} (**d**) and CML–$\phi_{Ganymede}$ (**e**) reproduced from [4] ("Reproduced with permission from Astronomy & Astrophysics, © ESO"). The colored lines corresponding to the boxes on the dynamic spectra, generated with the online Jupiter Probability Tool (https://jupiter-probability-tool.obspm.fr), represent the geometry at the time of each observation. When a color line crosses a white box, it indicates a high probability of detecting an emission induced by the corresponding satellite. Plots of panels (**d,e**) confirm the above identifications based on emission morphology.

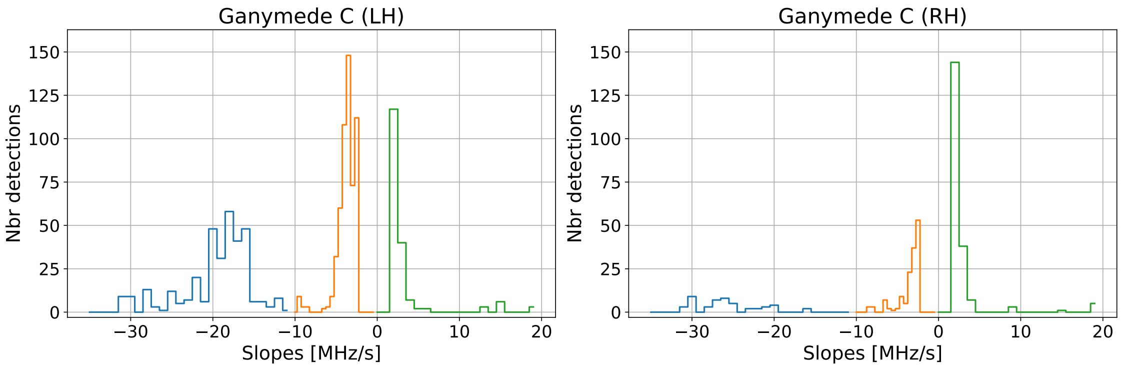

Supplementary Figure 4: **Histograms of slopes (drift-rates) for Ganymede-C emissions in both polarizations.** These histograms shows the propotion of drift-rates in LH polarization (left) and RH polarization (right), for the bursts detected in the Ganymede "C" box of Figure 1b in the paper. The color code is the same as in Figure 2.
