# Supplementary material for: Drifting discrete Jovian radio bursts reveal acceleration processes related to Ganymede and the main aurora
Source: Nat Commun. 2023 Oct 3;14:5981. doi: 10.1038/s41467-023-41617-8 (PMC10547699; doi:10.1038/s41467-023-41617-8)
Supplement: Supplementary file 3 — Description of Additional Supplementary Files [file 41467_2023_41617_MOESM3_ESM.docx]

File Name : Supplementary Data 1

Description : This file contains a sample of the output of the analysis of the data, with all the parameters listed in the Processing Method section of the Methods. As asked by the editors in the previous author guidances.
